# Work and family: associations with long-term sick-listing in Swedish women – a case-control study

**DOI:** 10.1186/1471-2458-7-287

**Published:** 2007-10-11

**Authors:** Hélène Sandmark

**Affiliations:** 1 Department of Public Health Sciences, Division of Occupational Medicine, Karolinska Institutet, Stockholm, Sweden; 2 Department of Health Sciences, Public Health Science, Örebro University, Örebro, Sweden

## Abstract

**Background:**

The number of Swedish women who are long-term sick-listed is high, and twice as high as for men. Also the periods of sickness absence have on average been longer for women than for men. The objective of this study was to investigate the associations between factors in work- and family life and long-term sick-listing in Swedish women.

**Methods:**

This case-control study included 283 women on long-term sick-listing ≥90 days, and 250 female referents, randomly chosen, living in five counties in Sweden. Bivariate and multivariate logistic regression analyses with odds ratios were calculated to estimate the associations between long-term sick-listing and factors related to occupational work and family life.

**Results:**

Long-term sick-listing in women is associated with self-reported lack of competence for work tasks (OR 2.42 1.23–11.21 log reg), workplace dissatisfaction (OR 1.89 1.14–6.62 log reg), physical workload above capacity (1.78 1.50–5.94), too high mental strain in work tasks (1.61 1.08–5.01 log reg), number of employers during work life (OR 1.39 1.35–4.03 log reg), earlier part-time work (OR 1.39 1.18–4.03 log reg), and lack of influence on working hours (OR 1.35 1.47–3.86 log reg). A younger age at first child, number of children, and main responsibility for own children was also found to be associated with long-term sick-listing. Almost all of the sick-listed women (93%) wanted to return to working life, and 54% reported they could work immediately if adjustments at work or part-time work were possible.

**Conclusion:**

Factors in work and in family life could be important to consider to prevent women from being long-term sick-listed and promote their opportunities to remain in working life. Measures ought to be taken to improve their mobility in work life and control over decisions and actions regarding theirs lives.

## Background

Since the end of the 1990s the number of people on long-term sick leave in Sweden has increased considerably, especially with regard to women. During the first years of the 21^st ^century, women accounted for more than 60 percent of the days for which cash benefit was paid out from the national sickness insurance due to long-term sick-listing. This concerns sick leave 60 days or more. Sick-listing has increased most for the 20–39 age group, and least for the 60–64 age group. The periods of sickness absence have also on average been longer for women than for men [1].

Yet the indicators have shown an improvement in the health of the Swedish working population during the years, with the exception of low-skilled women [2]. The high increase in sick-listing seems somewhat paradoxical in the view of the high standard of living and the high longevity in Sweden, and indicates that there is probably a complex causal relationship. It has been suggested that the high increase in sickness absence does not reflect a corresponding deterioration in health, or that the existing indicators of health might not be up to date. Exposures such as an increasingly tough working climate, reorganisations in the public sector, and new definitions of sickness and changed attitudes, have been suggested as possible reasons for the increase in long-term sickness absence [3,4]. As sickness absence is not identical with health status, but with inability to work, the mechanisms are not quite easy to establish [5]. It has been suggested that sickness absence could be an indicator of health in a population if it also includes social, psychological and physiological dimensions, as well as strictly medical dysfunctions [6].

Risk factors for sickness absence have been assessed in studies, but it remains aspects to be investigated. Among these are factors in working life and in family life, which are of special interest when investigating sickness absence in women. The fact that it is women who represent a great deal of the sickness absence cannot be explained by women staying home taking care of sick children to a greater extent than men, since the health insurance separates between staying at home taking care of a sick child and being home due to own sickness and inability to work.

Research has demonstrated that double exposure is a sometimes overlooked potential risk factor among women [7]. A determinant for women's long term sick leave might be that occupationally active women find themselves in a challenging psychosocial situation, because they still have the main responsibility for the family and household [8]. This has been shown in risk factors research, where high total workload from child care and occupational work was found to be associated with sickness absence in women [7,9-11]. However, Maastekasa (2000) added the enhancement theory, which means that multiple roles might have positive effects on health in the sense that more roles could be stimulating and positive for self-esteem, social identity and status [12,13]. Thus research has been somewhat conflicting and has not yet concluded if gainfully working women are at an increased risk for ill-health and sick-listing or the reverse.

It has been shown in a study that sickness absence in general is associated with the characteristics of the working environment, and the possibilities to be flexible in work and work performance [14]. The work inability can thus probably be determined by both health status and the demands from work. How job characteristics, such as flexibility in work life, part-time work, competence, industrial mobility – changes of jobs and professional positions – associate with health and sick leave have partly been investigated [15,16], but is not yet fully understood. For women the psychosocial working environment seems to be of more importance compared to men, regarding health and sickness absence [16-19].

The starting point for this study has been that risk factors for long-term sick listing in women are within a wide social context and that work ability and sick listing are complex concepts, which need to be further investigated. Further, it is crucial to bear in mind that the social insurance systems differ between countries, and that the mechanisms for sickness absence probably are closely related to how these systems function and vary.

The objective of this study was to investigate the associations between factors in work- and family life and long-term sick-listing in Swedish women. It is hypothesised that there are factors beyond those which are strictly connected to a medical diagnosis that could be associated with long-term sick-listing in Swedish women.

## Methods

### Health insurance in Sweden

The Swedish Insurance Office administrates social insurance, which is uniform throughout the country and funded by the Swedish state. Sickness compensation is possible for a significant reduction in work capacity caused by sickness or injury.

There is also a national insurance system, AGS (AvtalsGruppsjukförsäkringen *in Swedish*), which is a collective agreement between all parts of the Swedish Labour Union Confederation and the Swedish Employers' Confederation, which supplements benefits from the Social Insurance Office. It covers 2.5 millions of employees, mainly manual-, service- and healthcare workers in the public and private sectors. The register includes data on name, address, age, occupation, diagnosis and length of sick-listing period.

#### Study population and design

The association between long-term sick leave in Swedish women and factors related to occupational work and family life were investigated in a case-control study. The study base included totally 533 women aged 30 to 55 years, living in five counties in the middle part of Sweden between October 2003 and March 2004. These geographical areas represent a level just above the average in Sweden, regarding proportions of long-term sick-listing in women. They were selected as they include urban and rural areas, and a broad variation of demography, trade and industry.

Of the 533 women, 324 were randomly selected from the AGS register, and these constituted those who were long-term sick-listed; the cases. The criterion was being 30 to 55 years of age, living in the five counties, and having ongoing sickness absence ≥90 days. Sickness absence due to a diagnosis such as cancer, pregnancy complications, severe internal medical problems, severe coronary dysfuntions, stroke, severe psychiatric diagnosis or severe accidents, was not included. Women born outside the Nordic countries were not included. The most common reported causes of the sick leave among the cases in this study were musculoskeletal disorders, such as back- and neck pain, burn-out syndrome, less severe internal medical problems and coronary dysfunctions, fibromyalgia, depression, general pain, and stomach complaints. Of the randomly selected women 26 had started to work during the recent days and had not yet been taken out of the register, 15 were born outside the Nordic countries, had a diagnosis which was not included, or had moved outside the included geographical area recently. These women were not included in the study population as they did not fulfil the inclusion criteria. Thus 283 women were included, and of those 231 (82%) participated and answered a postal questionnaire.

The referents were randomly selected from the Swedish populations register. The random selection of 300 women was done from the same age-group as the cases (30 to 55), and from the same geographical areas. Those who were on long-term sick leave > 90 days or had an early retirement (n = 24), were born outside the Nordic countries or had recently moved outside the included geographical area (n = 26) were not included. Thus 250 women of the referents fulfilled the criteria to be included in the study and 194 (78%) chose to participate. The participation rate for cases and referents is 80%. An invitation letter was sent out about the study objectives, and thereafter informed consent was obtained from all participating subjects.

### Questionnaire and data collection

To gain an increased understanding of the contextual factors for long-term sick-listing, open-ended thematic interviews were performed with 25 women of the study population [20]. They were strategically selected to represent different ages, occupations, and geographical areas. The interviews gave an increased understanding of what could be included in the background of the women's long-term sick-leave regarding factors related to working life and family life and physical and mental dysfunctions [20]. Subsequently a questionnaire was elaborated on the basis of these results. Background questions were age, civil status, age at first child, number of children, present and past work, employers, and workplaces and part-time and full-time work during the years. Two questions on physical and mental demands at work have been used in earlier scientific research [21]. Work-related questions connected to the demand-control model were included in the questionnaire [22]. Questions on self-rated competence for work tasks, feedback from supervisors and work mates, and impact of organisational changes were also included. Regarding responsibility for own children and taking care of own children, and responsibility for domestic work and performance of domestic work during the years, four new questions were constructed. The interviewed women participated in the validation and in the testing of reliability of the questionnaire. Three weeks after the interviews they answered the postal questionnaire, and the answers were qualitatively validated with the interviews. Three weeks after the women had filled in the questionnaire the first time they got the same postal questionnaire once again. Two questions were not reliable and were therefore removed.

The questionnaire was then sent by mail to the entire study population. In case of missing answers the questionnaire was sent once again or a supplementary telephone interview was done.

### Classification of occupational positions

The occupational titles were classified into broadly similar categories in order to make sure the case-group and control-group were comparable. The Swedish National Standard for Classification of Skill Levels (SSYK 1996) was used for this purpose [23]. This national system is based on an international classification system, ISCO-88, and introduces the concept of skill, defined as the degree of complexity of constituent tasks and skill specialisation. Four skill levels are operationalised in terms of job-related formal training, which may be used to develop the skill level of persons who are to carry out such jobs.

The first skill level comprises those jobs only requiring primary education, such as cleaners, factory workers, and school meals assistants. The second skill level represents jobs requiring secondary education. These are, among others, assistant nurses, cashiers, and shop assistants. The third skill level represents jobs, which require a three-year university education. Such jobs are nurses, technicians and handling officers. The fourth skill level comprises jobs requiring four years or more of university education and an academic degree. These jobs are, for example, psychologist, personnel manager, and subject teacher in secondary education.

### Statistics

Odds ratios were used to estimate the strength of the associations between variables in work and private life and sick-listing ≥90 days. All information and exposures are based on self-reports. The cut-off points are shown in Table 1 and 2, respectively.

**Table 1:** Odds ratios^1 ^(OR) and 95% confidence intervals (CI) of the bivariate association between factors related to own children and sick-listing ≥ 90 days

**Variables**	**On Sick leave (N = 231)**	**Not on sick leave (N = 194)**	**Exposed cases (N)**	**OR (95%CI)**
Age at birth of first child	208	161	177	2.88 (1.82–4.74)
<28 years				
≥28 years				
Number of children	230	188	80	2.25 (1.42–3.54)
≥3 children				
0–2 children				
Main responsibility for own children	266	160	117	1.57 (1.04–2.41)
Yes/No				

^1^Odds Ratios adjusted for age

**Table 2:** Odds ratios (OR) ^1 ^and 95% confidence intervals (CI) of the bivariate association between self-reported work-related factors and sick-listing ≥ 90 days

**Variables**	**On sick leave (N = 231)**	**Not on sick leave (N = 194)**	**Exposed cases (N)**	**OR (95%CI)**
Number of employers during working life	230	192	123	1.75 (1.22–2.63)
<3 employers				
≥3 employers				
Number of employers during working life	50^2 ^(30–42 yrs)	36	37	4.85 (1.91–12.10)
<3 employers				
≥3 employers				
Number of employers during working life	33^3^	47	25	3.55 (1.43–9.31)
<3 employers				
≥3 employers				
Present employment status	221	174	19	0.62 (0.34–1.22)
Substitute				
Permanent				
Change of jobs/occupations during working life	127^2 ^(43–55 yrs)	58	44	2.75 (1.13–7.10)
<3 employers				
≥3 employers				
Change of workplaces during working life	103	102	60	1.45 (0.82–2.64)
<3 times				
≥3 times				
Part-time work	227	191	123	1.51 (1.03–2.23)
≥4 years				
<4 years				
Part-time work	227	192	49	2.24(1.43–3.78)
≥13 years				
<13 year				
Hours worked per week	229	193	110	0.88 (0.63–1.36)
>40 hours				
≤40 hours				
Lack of influence on working hours	231	194	105	2.76 (1.81–4.23)
No				
Yes/yes, partly				
Physical workload above capacity	227	194	56	3.91 (2.26–7.04)
Yes				
No/occasionally				
Too high mental strain in work tasks	228	190	49	4.06 (2.22–7.61)
Yes				
No/occasionally				
Workplace dissatisfaction	231	194	47	3.87 (2.10–3.43)
Dissatisfied				
Satisfied				
Work task dissatisfaction	219	187	38	4.31 (2.14–11.42)
Dissatisfied				
Satisfied				
Lack of competence for work tasks	167	147	23	4.54 (2.1–8.8)
Lack of competence				
Competent for work tasks				
Lack of appreciation at work	231	194	23	2.53 (1.14–5.83)
No appreciation Appreciation				
Bullying at work	103 (30–42 yrs)	102	16	2.29 (0.91–5.74)
Yes/No				
Bullying at work	50^2 ^(30–42 yrs)	36	12	4.41 (1.04–19.34)
Yes/No				
Organisational changes at work	204	162	53	2.02 (1.24–3.37)
Too demanding				
Do not care				
Overall dissatisfaction with working life	231	193	33	2.51 (1.28–4.92)
Very dissatisfied/rather dissatisfied				
Very satisfied/rather satisfied				

^1 ^Odds Ratios adjusted for age

^2 ^Women employed in the childcare and healthcare sectors

^3 ^Women in occupational skill level 3

The median age of the participating cases and controls, respectively, was calculated. The study population was divided into two age groups, 30 to 42 and 43 to 55 years of age in the bivariate analyses to control confounding from age. The bivariate calculations in Table 1 and 2 were done separately for each of the four different occupational skill levels, and also for those who had occupational titles indicating employment in health- and childcare. Where it differed between groups it is displayed in Table 2.

A multivariate logistic regression analysis was thereafter performed with sick-listing ≥90 days as outcome. Significant variables regarding the entire study population, and connected to gainful work was entered into a logistic regression model of analysis. Regarding part-time work, only the variable of ≥13 years of part-time work was included in the model. The variable; "Overall dissatisfaction with working life" was not included as it is a global and summarised variable.

The confidence intervals for all the odd ratios, in the bivariate analyses and in the multivariate logistic model were calculated for 95% confidence intervals.

The ethical review board at Karolinska Institutet approved the study, nr 03-542, and the sampling from the population register was also approved.

## Results

The women in the case-group had an ongoing sick-listing of 328 days (median), which ranged from 90 to 381 days. In the control-group 2% (n = 4) had an ongoing sicklisting that had lasted less than 90 days. The median age of the cases was 43 years of age, and 41 for the referents. The civil status for cases and referents was almost identical: 77% of the cases and 80% of the controls were married or cohabitants, 5% of the cases and 4% of the controls were divorced, and 13% of the cases and 12% of the controls were single.

The distribution of cases and referents in different labour-market sectors was about the same; 54% of the cases and 49% of the referents were working in the public sector, and 24% in each group worked in trade and industry. The remaining cases and controls were equally employed in state-owned enterprises, organisations, running their own businesses, or unemployed. Of the cases 3% and of the referents 8% were unemployed at the time for this investigation. The ten most common occupational titles were identical for cases and controls (Table 3).

**Table 3:** The ten most common occupational titles in order of precedence.

**Cases N = 231**	**Controls N = 194**
Assistant nurse	Assistant nurse
Nursing auxiliary	Teacher
Reg. nurse	Office worker
Cleaner	Nursing auxiliary
Child minder	Pre-school teacher
Teacher	Child minder
Pre-school teacher	Reg. nurse
Shop assistant	Shop assistant
Office worker	Cleaner
Personal assistant	Personal assistant

The bivariate analyses showed that long-term sick-listing was associated with number of children and main responsibility for the care of own children. The younger sick-listed women (30–42 years of age) had had their first child at an earlier age (Table 1).

Of the sick-listed women, 89% were permanently employed, compared with 78% of the referents. The sick-listed women had had lower industrial mobility during their working life, such as fewer changes of employers, work tasks and workplaces. The older sick-listed women (43–55 years of age) working in the childcare and healthcare sectors had changed jobs/occupations fewer times. Part-time work had been more common in the group of sick-listed women, and showed a stronger association if it had lasted for a longer time (Table 2).

The sick-listed women reported to a larger extent that their jobs were physically and mentally demanding, and that reorganisations at work were trying. There were also associations between reported lack of competence and dissatisfaction with workplace and workmates, and long-term sick-listing. The sick-listed women experienced less appreciation from supervisors, workmates and clients or patients. Bullying at work was associated with long-term sick listing among the younger women (30–42 years of age) working in the childcare and healthcare sectors (Table 2).

In Table 4 the logistic regression model shows that the strongest work-related risk factors were; reported lack of competence for work tasks, workplace dissatisfaction, mental and physical strain above capacity in work, numbers of employers during working life, earlier part-time work, and lack of influence on working hours.

**Table 4:** Multiple logistic regression model with sick-listing ≥ 90 days as outcome.

**Variables**	**Odds ratio (95% CI)**
Number of employers during working life	1.39 (1.35–4.03)
Change of workplaces during working life	0.29 (0.41–1.33)
Lack of influence on working hours	1.35 (1.47–3.86)
Part-time work ≥13 years	1.39 (1.18–4.03)
Physical workload above capacity	1.78 (1.50–5.94)
Too high mental strain in work tasks	1.61 (1.08–5.01)
Workplace dissatisfaction	1.89 (1.14–6.62)
Work task dissatisfaction	1.00 (0.35–2.71)
Lack of competence for work tasks	2.42 (1.23–11.21)
Lack of appreciation at work	0.51 (0.60–1.66)
Organisational changes at work perceived as demanding	0.92 (0.76–2.51)

Of the sick-listed women 93% reported that they wanted to return to working life, and 54% considered themselves able to work immediately if they could have a part-time job and 17% could work now if their work could be adjusted to them, or if they could change employer (26%). However, 67% of the sick-listed women believed that they would be working in 2 years time, compared with 87% of the referents.

## Discussion

The aim of this study was to investigate the associations between factors in working- and family life and long-term sick listing ≥90 days in Swedish women. The hypothesis that there are associations beyond those which are strictly connected to the medical diagnosis that could be associated with long-term sick leave in Swedish women was confirmed. In this study factors connected with occupational work and own children were studied, but the underlying disease or dysfunction was not further investigated. The results provide evidence that factors in working life connected with competence and influence, industrial mobility, dissatisfaction with work tasks, mental and physical strain above capacity, are associated with long-term sick-listing.

Long-term sick listing show associations with number of children, age at birth of first child and having the main responsibility for own children. The picture of a long-term sick-listed woman could be that she had had her first child rather early, and have more than two children, and took the main responsibility for the children over the years. The women in the reference group seemed to be in more equal family conditions. Thus long-term sick-listing in women was found to be associated with traditional family circumstances and inequality. Multiple demands from family and work could probably entail increased negative stress and be a challenge to women's health and well-being, and a determinant for long-term sickness absence. This is supported by studies, which found that those who have multiple roles and demands are more exposed to negative stress, resulting in physical and psychosocial dysfunctions [24-26]. Lundberg and co-workers (2003) found that during the 1990s the domestic workload, mainly connected with own children, increased for men and women, but to a greater extent for women [27]. In a study of personnel in the Swedish Post it was found that women with children and with exposure to domestic work had an increased risk for high sick leave [19].

Possibly the sick-listed women had experienced high demands, but not enough flexibility and influence at work. There might be an association with low decision latitude at work. Low influence on working hours is used in this study as a proxy for influence at work, and the women were comparable regarding occupations and jobs. The findings are in concordance with earlier studies, which have demonstrated that low job control is associated with sick leave, and a health hazard, especially in women [28,29]. Low job control and low social support at work has been found to increase the risk for mortality and sickness absence [30,31]. In recent research control over daily working hours has been found to be a health promoting factor, which might facilitate employees to combine a full-time job with the demands of domestic work [32].

Present employment status, such as being permanent employed or not, and hours worked per week did not differ between the investigated groups, but regarding part-time work the results differ and are also somewhat contradictory. In the analyses it was found that part-time work could possibly be potential risk factor for later long-term sick-listing, but on the other hand the sick-listed women reported that they could go back to working life if they could work part-time. This could be interpreted in the sense that doing part-time work earlier in life meant not giving wholehearted support to occupational work, leading to reduced opportunities for adapting to increased demands and the higher levels of competence required in working life later on. At present, when the women are on sick leave, part-time work could have a reverse effect and allow the individual to use at least part of her work ability. It has been concluded that it is mainly women with small children who want to decrease working hours [25]; however, we did not find that this demand was greater among younger women. Dellve et al (2006) found that part-time work was strongly related to long-term work ability among female home care workers. It is suggested that it could be a way of coping with demanding work, for example high workload [33]. A higher probability of illness has been observed in a study of those who had changed from full-time to part-time work although they were probably in a status of deteriorating health [34]. The associations seem to be complex and the causality is still unclear. The influence of part-time work on long-term sick leave probably depends on the time in life at which it appears, and the possibility of selection bias remains to be investigated.

For younger women in jobs within the healthcare and childcare sectors there was found an association with reported bullying at work from superiors or workmates. This could explain a later sickness absence, as bullying is serious and deeply affects the individual. It reflects a poor psychosocial climate at the workplace, as well as insufficient leadership. It has earlier been concluded that not only the victims, but also the observers at the workplace report negative stress due to the existence of bullying [35]. In a Finnish study, exposure to bullying was shown to be a risk factor for later sick-listing in women [36]. The study population was hospital staff, thus in many ways similar to our study population. An extensive Swedish study of health and sickness absence in women in the public sector, as well as a study of female personnel in a large Swedish company, showed associations between bullying at work and later sick-listing [19,37].

In the logistic model there were associations between dissatisfaction with the workplace and few employers over the years; and in the bi-variate analyses between lack of appreciation at work and an overall dissatisfaction with working life. These findings could be interpreted in different ways: One possible explanation could be that the sick-listed women to a larger extent were not in their most sought-after jobs or occupations, and that reported dissatisfaction therefore could have a negative impact on their work situation. Also, as the sick-listed women had more often been absent from work due to more pregnancies, and probably also for taking care of their children when they were ill, it is possible that they were not able to contribute satisfactorily at work or were not sufficiently updated to fulfil the demands at work. Earlier periods of absence from work due to children and childbirth could explain the sick-listed women's reported lack of competence. Increasing demands at the workplace during the past years probably require a continuous and steady presence to maintain skills and keep up to date. The sick-listed women in our study might not have been able to cope with these increasing demands, which were connected with reorganisations and staff cuts. They found reorganisations more demanding and trying. Being on long-term sick leave could be a strategy for women who are unable to handle the new and increasing demands at work due to downsizing and reorganisations. These findings are in concordance with those from other studies showing that all forms of organisational instability, downsizing or expansion are related to sickness absence and health hazards [3,18,40].

The results from this study must be interpreted in relation to the general situation on the labour market and how it has developed, especially during recent years when the number of people on sick leave has gone up. The increase in general job strain could certainly be a health hazard to those who have additional strain from family life. It is thus possible that the potential determinants for long-term sick-listing identified in this study could reflect deficiencies in changing work organisations. There has been increased sex segregation in the Swedish labour market during the 1990s and the beginning of this century. In areas where mostly women work, in the public sector, the conditions have deteriorated further, time pressure has increased, and there has been an increase in mental and physical workload, above all in labour-intensive services and in human services [3,19,38]. It has been concluded that organisational downsizing can deteriorate the psychosocial work environment and cause increased rates of sick leave in employees [39-41]. These circumstances can probably explain, at least to a certain extent, the rapid and extensive increase in long-term sick leave in women during the recent years.

### Methodological considerations

The study population was randomly selected from the AFA register and the Swedish populations register. Employees from different sectors of the labour market were thus included, especially those where women predominate. The findings could be generalised to a great deal of these women, especially those in the public sector and in the investigated age groups.

Certain medical diagnoses which have not increased during the past years were excluded in order to concentrate in those diagnoses which stand behind the recent increase in long-term sick-listing [42]. Immigrated women were not included in this study as it has been demonstrated that they differ from the native Swedish women regarding factors connected to sick leave and poor health [43]. There is no simple causal connection which can explain the differences, but it has been suggested that there is a reciprocal influence between health, work, and migration, which is more pronounced for women than for men [44]. This exclusion could cause an underestimation of potential risk for sick-listing in the women living in Sweden.

The data was collected at one occasion and therefore the causality is unclear. The direction of causality is somewhat clearer regarding data on number of children, age at birth of first child, number of employers and earlier jobs, but more unclear regarding reports of other factors in working life. As the study is based on retrospective self-reported data it is possible that the reporting could be dependent on the outcome, which is the sick leave itself, and a decreased health status among the cases. This is a limitation due to the design of this study, and some of the reported retrospective aspects of working life could have been influenced by the present long-term sick-leave. But this was what they reported at the time of our investigation and it reflects the circumstances when reporting. The factors investigated could hardly be estimated in any other way than by self-reports, unless the study would have had a prospective design. Therefore the conclusions should be drawn with care.

## Conclusion

The demands in contemporary working life have probably created health hazards in working women's situation. This study showed that long-term sick-listing in Swedish women is associated with factors in working life as well as in family life. Having the first child at an earlier age, having more children and taking the main responsibility for own children could possibly be determinants for later sick-leave. Low mobility in work life and part-time work could also be risk factors. Physical work load and mental strain above capacity were more common in long term sick-listed women, along with reported lack of competence for work tasks. The study provides some evidence that factors in occupational work, such as competence, presence and continuity at work, and mobility in working life, and factors related to own children and equal opportunities in family relations ought to be considered in health promoting activities.

## Competing interests

The author(s) declare that they have no competing interests.

## Authors' contributions

HS is the sole author.

## Pre-publication history

The pre-publication history for this paper can be accessed here:



## References

[B1] Försäkringskassan (The Swedish National Insurance Board) (2006). Social insurance statistics. http://statistik.forsakringskassan.se/portal/page?_pageid=47,41296&_dad=portal&_schema=PORTAL.

[B2] Statistiska centralbyrån, SCB (Statistics Sweden) (2004). Sjukfrånvaro och ohälsa i Sverige – en belysning utifrån SCB:s statistik 2004:3 (Sickness absence and ill-health in Sweden – in the light of statistics from SCB). http://www.scb.se/Grupp/Sjukfranvaro/_Dokument/Bfakta_analys.pdf.

[B3] Westerlund H, Ferrie J, Hagberg J, Jeding K, Oxenstierna O, Theorell T (2004). Workplace expansion, long-term sickness absence, and hospital admission. Lancet.

[B4] Vahtera J, Kivimäki M, Pentti J, Linna A, Virtanen M, Virtanen P, Ferrie JE (2004). Organisational downsizing, sickness absence, and mortality: 10-town prospective cohort study. BMJ.

[B5] Alexanderson K, Leijon M, Åkerlind I, Rydh H, Bjurulf P (1985). Epidemiology of sickness in a Swedish county in 1986 and 1987. A three year longitudinal study with focus on gender, age and occupation. Scand J Soc Med.

[B6] Marmot M, Feeney A, Shipley M, North F, Syme SL (1995). Sickness absence as a measure of health status and functioning: from the UK Whitehall II study. J Epidemiol Community Health.

[B7] Krantz G, Östergren PO (2001). Double exposure. The combined impact of domestic responsibilities and job strain on common symptoms in employed Swedish women. Eur J Public Health.

[B8] Hallsten L, Josephson M, Torgén M (2002). Performance based self-esteem: A driving force in burnout processes and its assessment. Arbete och Hälsa (Work and Health).

[B9] Voss M, Floderus B, Diderichsen F (2004). How do job characteristics, family situation, domestic work and lifestyle factors relate to sickness absence? A study based on Sweden Post. Occup Environ Med.

[B10] Åkerlind I, Alexanderson K, Hensing G, Leijon M, Bjurulf P (1996). Sex differences in sickness absence in relation to parental status. Scand J Soc Med.

[B11] Vistnes JP (1997). Gender differences in days lost from work due to illness. Labor Rel Rev.

[B12] Mastekaasa A (2000). Parenthood, gender and sickness absence. Soc Sci Med.

[B13] Bratberg E, Dahl S, Risa AE (2002). The double burden: Do combinations of career and family obligations increase sickness absence among women?. Eur Soc Rev.

[B14] Aronsson G, Gustafsson K, Dallner M (2000). Sick but yet at work: An empirical study of sickness presenteeism. J Epidemiol Community Health.

[B15] Bourbonnais R, Mondor M (2001). Job strain and sickness absence among nurses in the province of Quebec. Am J Ind Med.

[B16] Dellve L (2003). Explaining occupational disorders and work ability among home care workers. PhD thesis.

[B17] Ferrie JE, Shipley MJ, Stanfeld SA, Marmot MG (2002). Effects of chronic job insecurity and change in job security on self reported health, minor psychiatric morbidity, physiological measures, and health related behaviours in British civil servants: the Whitehall II study. J Epidemiol Community Health.

[B18] Kivimäki M, Feldt T, Vahtera J, Nurmi JE (2000). Sense of coherence and health: evidence from two cross-lagged longitudinal samples. Soc Sci Med.

[B19] Voss M, Floderus B, Diderichsen F (2001). Physical, psychosocial, and organisational factors relative to sickness absence: a study based on Sweden Post. Occup Environ Med.

[B20] Sandmark H, Renstig M (2004). Kvinnors sjukskrivning. Intervjuer med 25 långtidssjukskrivna kvinnor i Mellansverige (Women's sick-listing. Interviews with 25 sick-listed women in Mid-Sweden).

[B21] Tuomi K, Ilmarinen J, Jahkola A, Katajarinne L, Tulkki A (1998). Work Ability Index. Occupational Health Care No 19, 2nd revised edn.

[B22] Karasek C, Theorell T (1990). Healthy work.

[B23] Statistiska centralbyrån, SCB (Statistics Sweden) (2006). Standard för svensk för svensk yrkesklassificering (Standard for Swedish classification of occupations). http://www.scb.se/templates/Standard_36492.asp.

[B24] Scharlach AE (2001). Role strain among working parents: implications for workplace and community. Community Work Family.

[B25] Nordenmark M (2004). Balancing work and family demand. Do increasing demands increase strain? A longitudinal study. Scand J Publ Health.

[B26] Hallsten L, Bellagh K, Gustafsson K (2002). Utbränning i Sverige – en populationsstudie (Burnout in Sweden – a national survey). Arbete och Hälsa (Work and Health).

[B27] Lundberg U, Krantz G, Berntsson L (2003). Total arbetsbörda, stress och muskelvärk i ett genusperspektiv (Total workload, stress and muscle pain from a gender perspective). J Soc Med.

[B28] Ala-Mursula L, Vahtera J, Linna A, Pentti J, Kivimaki M (2005). Employee work time control moderates the effects of job strain and effort-reward imbalance on sickness absence: the 10-town study. J Epidemiol Community Health.

[B29] Steptoe A, Willemsen GJ (2004). The influence of low job control on ambulatory blood pressure and perceived stress over the working day in men and women from the Whitehall II cohort. J Hypertens.

[B30] Bosma H, Peter R, Siegrist J, Marmot M (1998). Two alternative job stress models and the risk for coronary disease. Am J Public Health.

[B31] Vahtera J, Kivimäki M, Pentti J, Theorell T (2000). Effect of change in the psychosocial work environment on sickness absence: a seven year follow up of initially healthy employees. J Epidemiol Community Health.

[B32] Ala-Mursula L, Vahtera J, Kouvonen A, Vaananen A, Linna A, Pentti J, Kivimaki M (2006). Long hours in paid and domestic work and subsequent sickness absence: does control over daily working hours matter?. Occup Environ Med.

[B33] Dellve L, Karlberg C, Allebeck P, Herloff B, Hagberg M (2006). Macro-organizational factors, the incidence of work disability, and work ability among the total workforce of home care workers in Sweden. Scand J Public Health.

[B34] Filippi M, Villosio C, Mamo C, Costa G (2005). Study of the relationship among work and demographic characteristics, sickness absence and occupational mobility. Med Lav.

[B35] Vartia MA (2001). Consequences of workplace bullying with respect to the well-being of its targets and the observers of bullying. Scan J Work Environ Health.

[B36] Kivimäki M, Elovainio M, Vahtera J (2000). Workplace bullying and sickness absence in hospital staff. Occup Environ Med.

[B37] Vingård E, Lindberg P, Josephson M, Voss M, Heijbel B, Alfredsson L, Stark S, Nygren Å (2005). Long-term sick-listing among women in the public sector and its associations with age, social situation, lifestyle, and work factors: A three-year follow-up study. Scan J Publ Health.

[B38] Härenstam A, MOA Research Group (2005). Different development trends in working life and increasing occupational stress require new work environment strategies. Work.

[B39] Westerlund H, Theorell T, Alfredsson L (2004). Organisational instability and cardiovascular risk factors in white-collar employees. Eur J Public Health.

[B40] Head J, Kivimäki M, Martikainen P, Vahtera J, Ferrie JE, Marmot MG (2006). Influence of change in psychosocial work characteristics on sickness absence: the Whitehall II study. J Epidemiol Community Health.

[B41] Vathera J, Kivimäki M, Pentti J, Theorell T (2000). Effect of change in the psychosocial work environment on sickness absence: a seven year follow up of initially healthy employees. J Epidemiol Community Health.

[B42] RFV redovisar (2003). Långtidssjukskrivna – egenskaper vid 2003 års RFV-LS-undersökning. Riksförsäkringsverket; (RFV reports. Long-term sick-listing – characteristics in RFV-LS, 2003 – investigation).

[B43] Copper H (2002). Investigating socio-economic explanations for gender and ethnic inequalities in health. Soc Sci Med.

[B44] Akhavan S, Bildt CO, Franzen EC, Wamala S (2004). Health in relation to unemployment and sick leave among immigrants in Sweden from a gender perspective. J Immigr Health.

